# Predicting Postoperative Stress Urinary Incontinence After Prolapse Surgery via Machine Learning and Regression Models: Development and Validation Study

**DOI:** 10.2196/76021

**Published:** 2025-11-03

**Authors:** Minna Su, Shuyu Wang, Xiaochun Liu

**Affiliations:** 1 Gynecology and Obstetrics Department, Third Hospital of Shanxi Medical University, Shanxi Bethune Hospital, Shanxi Academy of Medical Sciences, No. 99 Longcheng Street, Xiaodian District, Taiyuan, 030000, China, 86 13934226668

**Keywords:** prediction model, pelvic organ prolapse, de novo stress urinary incontinence, machine learning, support vector machine

## Abstract

**Background:**

Pelvic organ prolapse (POP) and stress urinary incontinence (SUI) often concurrently exist. The incontinence in some patients with POP resolves after POP surgery, but it persists in others. Some patients without SUI before surgery may develop de novo SUI. It is unclear whether a concomitant anti-incontinence procedure should be performed at the time of POP surgery to prevent postoperative incontinence. A prediction model is needed to guide clinical decision-making.

**Objective:**

This study aimed to analyze the risk factors and develop prediction models for SUI after POP surgery based on machine learning to provide new tools for evaluating and predicting postoperative SUI.

**Methods:**

Sample size calculation was performed using the Riley 4-step method. Data of patients undergoing prolapse surgery in Shanxi Bethune Hospital were prospectively collected from August 2022 to February 2025 and were retrospectively collected from January 2021 to August 2022. General clinical data, relevant laboratory test results, urodynamic examination findings, and pelvic floor ultrasound findings were collected. Lasso regression, univariate analysis, and logistic analysis were used to screen the predictors of SUI after prolapse surgery. Data were split randomly in a 7:3 ratio into training and validation sets. The training set was used to develop the prediction model involving Lasso regression, random forest, support vector machine (SVM), extreme gradient boosting (XGBoost), classification and regression tree (CART), and logistic regression, and the validation set was used for internal verification. The final implementation was achieved by developing a Shiny-based application for model deployment.

**Results:**

A total of 286 patients were enrolled in this study, and 91 patients had postoperative SUI. The following 6 risk factors were identified through univariate, logistic, and Lasso regression analyses: preoperative SUI, urge urinary incontinence, urodynamic occult SUI, anti-incontinence surgery, genital hiatus, and anterior colporrhaphy. Five prediction models were constructed by using logistic regression, random forest, XGBoost, SVM, and CART. Based on a comprehensive evaluation of model discrimination, calibration, and clinical utility, the SVM model demonstrated optimal overall performance, with an area under the curve of 0.821 in the training set and 0.846 in the validation set.

**Conclusions:**

This study developed 5 prediction models for postoperative SUI following prolapse surgery, which demonstrated good performance in internal validation. Among them, the SVM prediction model appeared to be the most promising. However, further external validation data are required to assess its generalizability. This model has the potential to become a high-quality clinical risk prediction tool for postoperative SUI in patients with prolapse, guiding clinical decisions on whether concurrent prolapse and incontinence surgeries are necessary.

## Introduction

Pelvic organ prolapse (POP) and stress urinary incontinence (SUI) are the 2 most common types of female pelvic floor dysfunctions. POP refers to the abnormal positioning and dysfunction of female pelvic organs (vagina, bladder, uterus, or rectum) caused by weak pelvic floor supportive tissues, which can lead to significant physical and emotional discomfort [1]. SUI refers to the involuntary leakage of urine during physical exertion, coughing, or sneezing (increased abdominal pressure) [2]. SUI occurs in 50% of patients with POP [3]. After POP surgery, 14.3%‐32.5% of patients have SUI, likely due to either uncorrected bladder neck defects after prolapse repair or the unmasking of pre-existing SUI symptoms that were previously concealed by the kinking of the lower urinary tract during prolapse [4]. To reduce the risk of postoperative SUI, prolapse surgery and incontinence surgery can be performed concomitantly. However, concomitant operations have caused many patients who would not have SUI to undergo an unnecessary operation, and the current common operation for the treatment of SUI, midurethral sling, is often accompanied by many postoperative adverse reactions [5,6]. There is still no consensus on whether patients with coexisting POP and SUI should undergo staged surgeries or a concomitant anti-SUI surgery.

The risk factors for urinary incontinence after prolapse include point Ba (the lowest point of prolapse between point Aa anteriorly and the vaginal apex), preoperative SUI, preoperative occult SUI, diabetes, sacral colpopexy, etc [7,8]. A risk prediction model is a statistical model that estimates the probability of an individual having a certain outcome according to the risk factors of the disease. Predictive models for assessing SUI risk after prolapse surgery can effectively aid patient decision-making. Jelovsek et al [9] used data from the OPUS (Outcomes Following Vaginal Prolapse Repair and Midurethral Sling) trial to construct a predictive model for de novo SUI in patients with POP who do not have SUI before surgery, and the area under the curve (AUC) of this model was 0.72. Based on data from the CUPIDO (Concomitant Surgery and Urodynamic Investigation in Genital Prolapse and Stress Incontinence: A Diagnostic Study Including Outcome Evaluation) trial, van der Ploeg et al [10] developed a prediction model for postoperative SUI in patients with POP who did or did not have preoperative SUI. Compared with the model constructed using OPUS data, the predictive factor stress test was added. This prediction model has an AUC of 0.79. An article published in BJOG reviewed 1142 patients who underwent POP surgery from 2 tertiary hospitals in South Korea and developed a predictive model for SUI after prolapse surgery, with an AUC of 0.74 [11].

With the development of artificial intelligence in recent years, machine learning has become an important method in the field of predictive model construction. Compared to traditional methods, machine learning methods have higher predictive accuracy and are commonly used in the medical field to predict various outcomes [12,13]. In a study conducted by Peking Union Medical College Hospital in China, a predictive model was constructed for the first time using machine learning methods (logistic regression, random forest, and extreme gradient boosting [XGBoost]) [14].

Previous studies were retrospective, and the machine learning predictive model approach used only random forest and XGBoost methods. No prospective studies have been conducted to establish a predictive model for SUI after POP surgery. This study prospectively followed patients with POP and retrospectively collected data from additional POP surgical cases to increase the sample size. Detailed clinical parameters were obtained, including pelvic ultrasound findings, hematologic markers, and operations. Machine learning prediction models for postoperative SUI in patients with POP (with or without SUI) were developed and validated to enable more accurate risk stratification and clinical decision-making.

## Methods

### Study Design and Population

This study collected data prospectively from August 2022 to February 2025 and retrospectively from January 2021 to August 2022 from patients undergoing POP surgery at Shanxi Bethune Hospital in China. The study enrolled patients with Pelvic Organ Prolapse Quantification (POP-Q) stages II-IV, regardless of preoperative SUI status, who had undergone prolapse surgery or were scheduled to undergo prolapse surgery. The follow-up period was at least 6 months. The exclusion criteria were prior prolapse surgery and postoperative urinary tract infection.

The Riley 4-step method, specifically used in clinical prediction models, was adopted for sample size calculation [15]. This study developed binary prediction models with an anticipated 8 predictor parameters. For internal validation, the anticipated shrinkage level was set at 0.9. The postoperative SUI incidence rate at our hospital is approximately 30%. With an assumed model C-statistic of 0.8, we calculated the sample size requirement using the R package “pmsampsize.” The results showed that the minimum sample size required for the new model was 276.

### Data Collection

Considering SUI risk factors in previous studies, relevant clinical characteristics of patients were collected, including age, height, weight, BMI, parity, vaginal delivery count, cesarean section count (due to low positive rates, this item was finally excluded), menopausal status, surgical age minus menopausal age, smoking history, type of medical insurance (Employee Basic Medical Insurance/Resident Basic Medical Insurance), educational background, preoperative estrogen supplement, postoperative estrogen supplement, preoperative SUI, preoperative urge urinary incontinence (UUI), hypertension, diabetes, comorbidities, POP-Q points and grade, biochemical indicators (albumin, liver function tests, and lipid profile; renal function tests were excluded due to low positive rates), cystocele Green type, residual urine, urodynamic occult stress urinary incontinence (UOSUI), urine leakage point pressure, anterior colporrhaphy performed (yes or no), perineal body reconstruction performed (yes or no), anti-SUI procedure performed (yes or no), hysterectomy performed (yes or no), and prolapse surgery method. Since urine leak point pressure only exists in patients with urodynamic stress incontinence, it is not conducive to model construction, and thus, it was not considered. Surgical methods for prolapse included colpocleisis, sacrospinous ligament fixation, round ligament suspension, high uterosacral ligament suspension, transvaginal mesh implantation, Manchester procedure, sacrocolpopexy, and lateral abdominal wall suspension. If only hysterectomy or anterior/posterior colporrhaphy was performed, the patient was coded as 0 in surgical evaluations. The operation for SUI was midurethral sling. Preoperative urinary incontinence status was determined through retrospective review of medical records combined with patient recall during postoperative follow-up. Postoperative SUI was defined as the reporting of bothersome SUI symptoms during any follow-up visit. Patients were followed up via outpatient clinic visits or telephone interviews.

### Data Preprocessing

Smoking history was excluded because of more than 10% missing data and a low positive rate (2 out of 286). Educational background was excluded because of more than 10% missing data. The remaining missing values were multi-interpolated using the R package “mice.” Patients with missing postoperative SUI outcomes were ultimately excluded from the study. Therefore, all postoperative SUI outcomes were based on the actual postoperative follow-up.

### Model Development

Data were split randomly in a 7:3 ratio into a training set and a validation set. We ensured that there were no statistically significant differences in baseline characteristics between the training set and the validation set. The training set was used to develop the model, and the validation set was used for internal validation.

We performed 5-fold cross-validated Lasso (least absolute shrinkage and selection operator) regression using the R package “glmnet” to screen for predictive variables. Lasso regression was implemented with L1 regularization (tuning parameter λ) to select variables via coefficient shrinkage and prevent overfitting, thereby improving generalization. Multivariate logistic regression analysis was performed using the variables obtained by Lasso regression to construct a nomogram prediction model.

The decision tree model in this study was constructed based on the classification and regression tree (CART) method developed in 1984 [16]. CART analysis is a nonparametric, nonlinear method that generates binary trees by feature selection based on Gini index minimization criteria.

Random forest and XGBoost are both integrated machine learning methods based on decision trees. The random forest prediction model is created based on the bagging algorithm and can be used for both classification and regression analyses [17]. We developed the random forest model using bootstrap resampling techniques. We assessed feature importance in 2 metrics: mean decrease accuracy (based on permutation) and mean decrease Gini (based on Gini impurity). Considering the direct relevance of mean decrease accuracy to model prediction accuracy, its stronger robustness, and its better interpretability in clinical contexts [18,19], we ultimately selected the top 8 most predictive features based on the ranking results of mean decrease accuracy to construct the final model. XGBoost develops the model by gradually adding decision trees, each of which resolves the error weakness of the previous one to achieve an accurate classification effect [20]. The XGBoost model incorporates regularization terms based on tree complexity, effectively reducing overfitting and thereby enhancing generalization performance. XGBoost was configured to utilize the top 8 most important variables. The optimal parameters were selected via hyperparameter optimization, with additional L2 regularization (lambda) and L1 regularization (alpha) penalties incorporated to mitigate overfitting.

Support vector machine (SVM) is a powerful and versatile machine learning method that excels at both classification and regression tasks. Based on the limited sample information, it seeks the best compromise between the complexity and learning ability of the model. Its objective is to identify the optimal hyperplane that separates data into 2 distinct classes, where support vectors represent the critical data points that maximize the margin between the 2 classes. In this study, 4 SVM methods were employed: linear SVM, polynomial SVM, radial basis SVM, and sigmoid SVM. The kernel with the best performance was finally selected to develop the model [21].

The “glmnet,” “rms,” “random forest,” “XGBoost,” “e1071,” and “rpart” packages in R software (R Foundation for Statistical Computing) were used to construct logistic regression, random forest, XGBoost, SVM, and CART prediction models.

### Model Evaluation

The models were internally validated using validation sets. AUC, accuracy, precision, sensitivity, specificity, and *F*_1_-score were used to evaluate the discrimination of the models. The prediction model calculated the AUC based on probability outputs. AUC values approaching 1.0 were considered to indicate superior predictive performance for postoperative SUI, while AUC values near 0.5 were considered to suggest limited clinical utility.

Model calibration was evaluated by both the Brier score and calibration curves. The Brier score quantifies prediction accuracy, with values approaching 0 indicating perfect prediction and values nearing 1 representing the poorest performance. Calibration curves were used to visualize the agreement between predicted probabilities and actual frequencies, where perfect calibration follows the 45-degree line.

Decision curve analysis (DCA) was used to evaluate the application value of the model in practical clinical decision-making and provide guidance for clinical decision-making.

### Statistical Analysis

All data processing and statistical analyses were performed using R software (version 4.4.2). Numerical variables have been presented as mean (SD) for normally distributed data (analyzed with the Student *t* test) or median (IQR [P25, P75]) for nonnormally distributed data (analyzed with the Mann-Whitney *U* test). Categorical variables have been expressed as frequency (n) and were compared using chi-square tests.

### Ethical Considerations

The study was approved by the Shanxi Bethune Hospital Medical Ethics Review Committee (YXLL-2025‐072) and was performed in accordance with the principles of the Declaration of Helsinki. Written informed consent was obtained from each participant before taking measurements. All data were anonymized, and the study was conducted without offering any financial or material incentives to the participants.

## Results

### Clinical Characteristics

**Figure 1.**
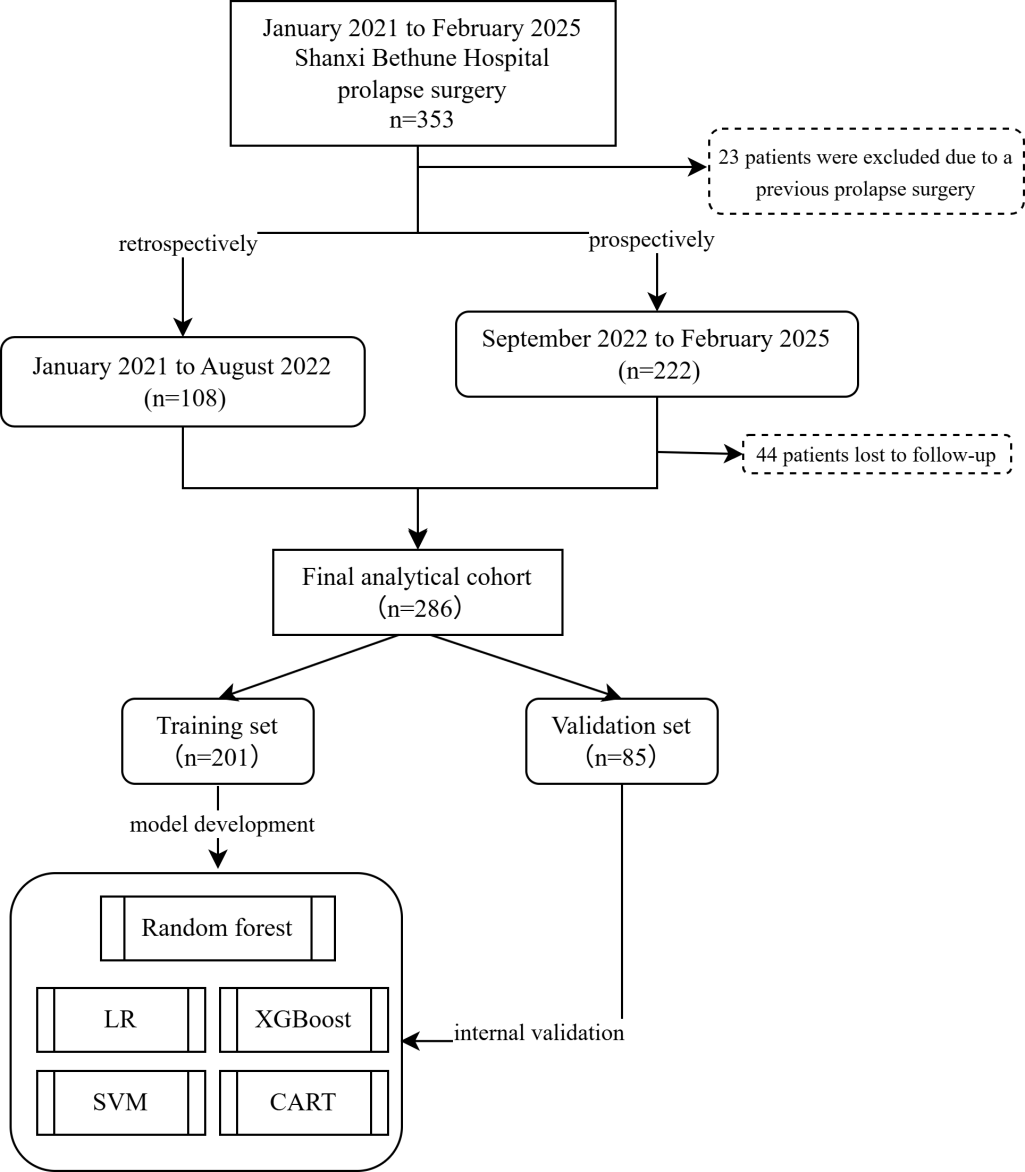
Flowchart of patient selection and model development and validation. CART: classification and regression tree; LR: logistic regression; SVM: support vector machine; XGBoost: extreme gradient boosting.

Between January 2021 and February 2025, 353 patients underwent prolapse surgery at Shanxi Bethune Hospital. Of these patients, 23 were excluded due to a history of previous prolapse surgery, leaving 108 retrospectively enrolled patients from January 2021 to August 2022 and 222 prospectively recruited patients from September 2022 to February 2025. However, 44 patients were lost to follow-up. Thus, the final analytical cohort included 286 patients (Figure 1). These patients were randomly allocated to the training (n=201) and validation (n=85) sets in a 7:3 ratio, with no significant differences in baseline characteristics between the groups (all *P*>.05).

A total of 286 patients undergoing prolapse surgery were included, and of these patients, 91 (31.8%) developed postoperative SUI, including 27 (9.4%) with de novo SUI and 64 (22.4%) with persistent SUI. In univariate analysis, preoperative SUI, UUI, UOSUI, and longer genital hiatus were identified as significant predictors of postoperative SUI, while concomitant anti-SUI surgery was identified as a protective factor. Variables with *P*≤.05 in univariate analysis are presented in Table 1.

Multivariate logistic regression analysis was performed for these 5 variables (Table 2). The results showed that preoperative SUI, UOSUI, length of genital hiatus, and concomitant anti-SUI surgery were influential factors for SUI after prolapse surgery.

**Table 1.** The results of univariate analysis.

Variable	Total (N=286)	Postoperative SUI^a^ (−) (n=195)	Postoperative SUI (+) (n=91)	*t* test (*df*)	Chi-square (*df*)	*P* value
Gh^b^, mean (SD)	5.44 (0.92)	5.28 (0.93)	5.78 (0.81)	−4.42 (284)	—^c^	<.001
SUI, n (%)				—	59.79 (1)	<.001
Absent	175 (61.2)	149 (76.4)	26 (28.6)			
Present	111 (38.8)	46 (23.6)	65 (71.4)			
UUI^d^, n (%)				—	7.50 (1)	.006
Absent	244 (85.3)	174 (89.2)	70 (76.9)			
Present	42 (14.7)	21 (10.8)	21 (23.1)			
UOSUI^e^, n (%)				—	13.92 (1)	<.001
Absent	255 (89.2)	183 (93.8)	72 (79.1)			
Present	31 (10.8)	12 (6.2)	19 (20.9)			
Anti-SUI surgery, n (%)				—	4.25 (1)	.04
No	267 (93.4)	178 (91.3)	89 (97.8)			
Yes	19 (6.6)	17 (8.7)	2 (2.2)			

a SUI: stress urinary incontinence.

b Gh: genital hiatus.

c Not applicable.

d UUI: urge urinary incontinence.

e UOSUI: urodynamic occult stress urinary incontinence.

**Table 2.** Multivariate logistic regression analysis.

Variable	β	SE	Z	*P* value	OR^a^ (95% CI)
Intercept	−5.17	1.07	−4.82	<.001	0.01 (0.00-0.05)
Gh^b^	0.60	0.18	3.26	.001	1.83 (1.27-2.62)
SUI^c^					
Absent	—^d^	—	—	—	1.00 (reference)
Present	2.16	0.32	6.77	<.001	8.71 (4.66-16.30)
UUI^e^					
Absent	—	—	—	—	1.00 (reference)
Present	0.76	0.42	1.78	.07	2.13 (0.93-4.89)
UOSUI^f^					
Absent	—	—	—	—	1.00 (reference)
Present	1.12	0.54	2.06	.04	3.05 (1.06-8.80)
Anti-SUI surgery					
No	—	—	—	—	1.00 (reference)
Yes	−3.03	0.84	−3.60	<.001	0.05 (0.01-0.25)

a OR: odds ratio.

b Gh: genital hiatus.

c SUI: stress urinary incontinence.

d Not applicable.

e UUI: urge urinary incontinence.

f UOSUI: urodynamic occult stress urinary incontinence.

### Model Development

#### Logistic Regression Predictive Model

**Figure 2.**
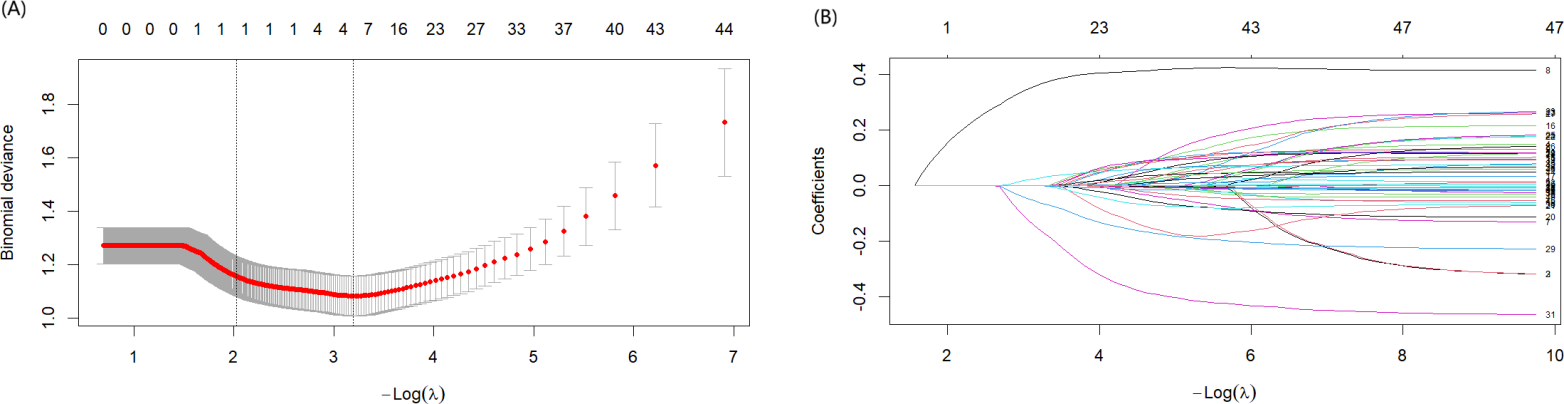
Lasso regression for filtered predictors. (A) Cross-validation error; (B) Coefficient paths.

Lasso regression was used to identify nonzero coefficient predictors from both the postoperative SUI and non-SUI groups, thereby refining the risk factors for postoperative SUI complications (Figure 2). The optimal λ value (0.041) was selected through 5-fold cross-validation, and 4 nonzero coefficient predictors were selected, including preoperative SUI, anterior colporrhaphy, anti-SUI surgery, and length of genital hiatus.

**Figure 3.**
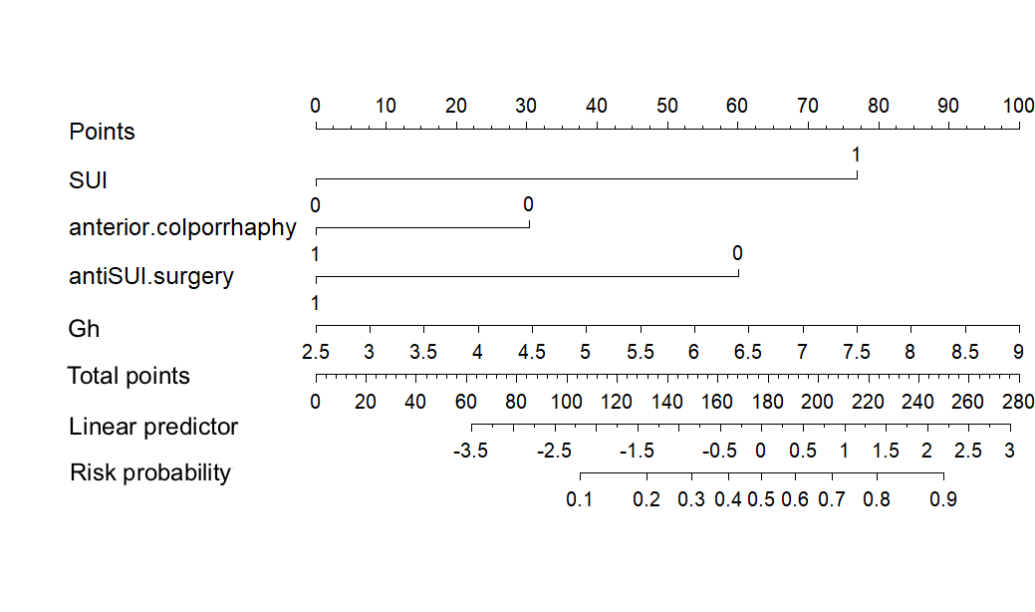
Logistic regression model nomogram. Gh: genital hiatus; SUI: stress urinary incontinence.

Subsequently, the risk factors selected by Lasso regression were incorporated into the logistic regression predictive model, with the corresponding nomogram presented in Figure 3. The model demonstrated an AUC of 0.817 (95% CI 0.755‐0.880) in the training set, maintaining robust discrimination upon internal validation (AUC=0.869, 95% CI 0.791‐0.947).

#### Random Forest Predictive Model

**Figure 4.**
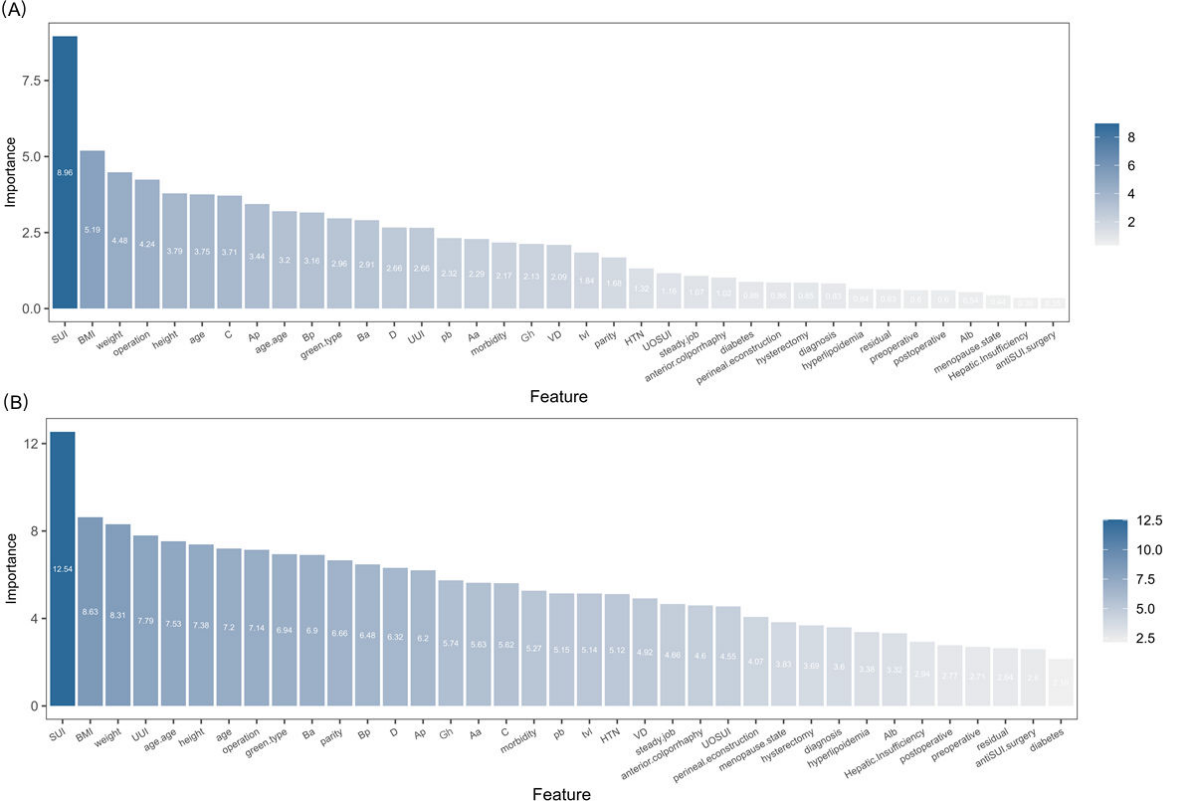
Feature importance plot of the random forest model. (A) Feature importance plot of the random forest model based on the Gini index; (B) Feature importance plot of the random forest model based on accuracy. HTN: hypertension; SUI: stress urinary incontinence; UOSUI: urodynamic occult stress urinary incontinence; UUI: urge urinary incontinence; VD: vaginal delivery.

A preliminary random forest model was constructed with 38 variables, and the importance of the variables was calculated, as shown in Figure 4. We chose to include the top 8 features (SUI, BMI, weight, UUI, age at menopause, height, age, and operation) as ranked by mean decrease accuracy, since this measure more directly reflects a feature’s contribution to classification performance and mitigates the evaluation biases associated with the Gini index. To optimize model performance, hyperparameter tuning was conducted through cross-validation to determine the optimal *mtry* value. Subsequently, the out-of-bag error was used to identify the ideal number of decision trees (ntree) for ensuring model stability and convergence. After parameter adjustment and bootstrap resampling (n=500 iterations), the parameters of the final random forest prediction model were set as follows: max_depth=5, min_samples_split=10, min_samples_leaf=5, mtry=3, ntree=146, maxnodes=20, and nodesize=5. The random forest model showed good differentiation in the training set (AUC=0.855, 95% CI 0.796‐0.914), but the AUC dropped to 0.731 (95% CI 0.604‐0.858) in the internal validation.

#### XGBoost Model

**Figure 5.**
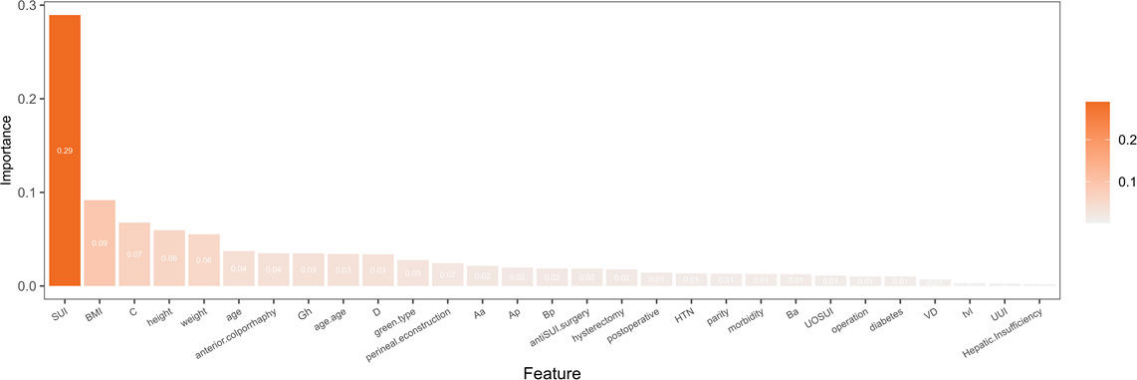
Feature importance of the extreme gradient boosting model based on accuracy. HTN: hypertension; SUI: stress urinary incontinence; UOSUI: urodynamic occult stress urinary incontinence; UUI: urge urinary incontinence; VD: vaginal delivery.

The regularization and early stop methods were used to reduce the overfitting of the model, and the optimal parameters and top 8 variables of importance were selected to build the XGBoost prediction model. The importance of accuracy based on the preliminary XGBoost model is shown below (Figure 5). After hyperparameter tuning by the train() function and 5-fold cross-validation, the final XGBoost model demonstrated enhanced predictive performance with the following configuration: eta=0.01, max_depth=3, gamma=0.1, colsample_bytree=0.8, min_child_weight=1, subsample=0.8, lambda=3, alpha=.5, early_stopping_rounds=10, and nrounds=200. The XGBoost prediction model had an AUC of 0.899 (95% CI 0.856‐0.943) and an AUC of 0.783 (95% CI 0.680‐0.886) in the training set.

#### CART Model

**Figure 6.**
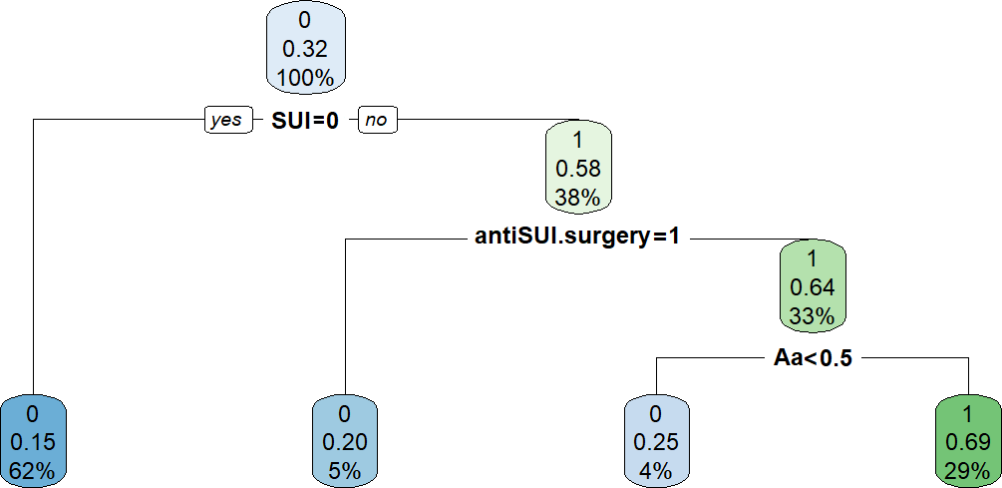
Decision tree. SUI: stress urinary incontinence.

The CART model, after pruning according to the optimal complexity parameters (0.625), is shown in Figure 6, illustrating the final classification tree used to predict the occurrence of postoperative SUI. The CART model contained 3 branches based on 3 key predictive factors: preoperative SUI status, concomitant anti-SUI procedure, and POP-Q point C, which simplified postoperative SUI assessment. The model directed “yes” responses to the left node and “no” responses to the right node. For patients without preoperative SUI (representing 62% of the cohort), the postoperative SUI probability was 0.15. The AUC of this decision tree model was 0.764 (95% CI 0.695‐0.832). In the validation set, the AUC was 0.782 (95% CI 0.677‐0.886). The model exhibited minimal overfitting.

#### SVM Model

After hyperparameter tuning of the 4 kernel functions (linear kernel, radial base kernel, polynomial kernel, and sigmoid kernel), their accuracy, precision, recall, specificity, and *F*_1_-scores were calculated, and the findings are summarized in Table 3. We observed that the sigmoid kernel predicted all samples as class 0. This phenomenon may be attributed to a mismatch between the sigmoid kernel function and the characteristics of the dataset in our study. Under the optimized hyperparameters (gamma=0.1; coefficient 0=3), the decision function values for all samples were negative (ranging from −0.92 to −4.05) and were classified as class 0 according to the decision rule of SVMs, resulting in the model’s failure to form an effective classification boundary. Consequently, the precision, recall, and *F*_1_-score of the sigmoid model were considered invalid.  

The preliminary analysis found that the polynomial kernel SVM classifier had better performance than the other 3 kernel classifiers (Table 3), and thus, polynomial kernel modeling was selected. Finally, the SVM prediction model was developed with 4 variables obtained by Lasso regression. The AUC of the final SVM model was 0.821 (95% CI 0.760‐0.883), and the validation set AUC was 0.846 (95% CI 0.757‐0.936).

**Table 3.** Evaluation of support vector machine kernels.

Model type	Accuracy, %	Precision, %	Recall, %	Specificity, %	*F*_1_-score, %
Linear kernel	72.94	62.96	56.67	81.82	59.62
Polynomial kernel	78.82	66.67	66.67	84.48	66.67
Radial base kernel	76.47	51.85	66.67	79.69	58.33
Sigmoid kernel	68.24	—^a^	—	100.00	—

a Not applicable.

### Comparison of the 5 Models and Applications

**Figure 7.**
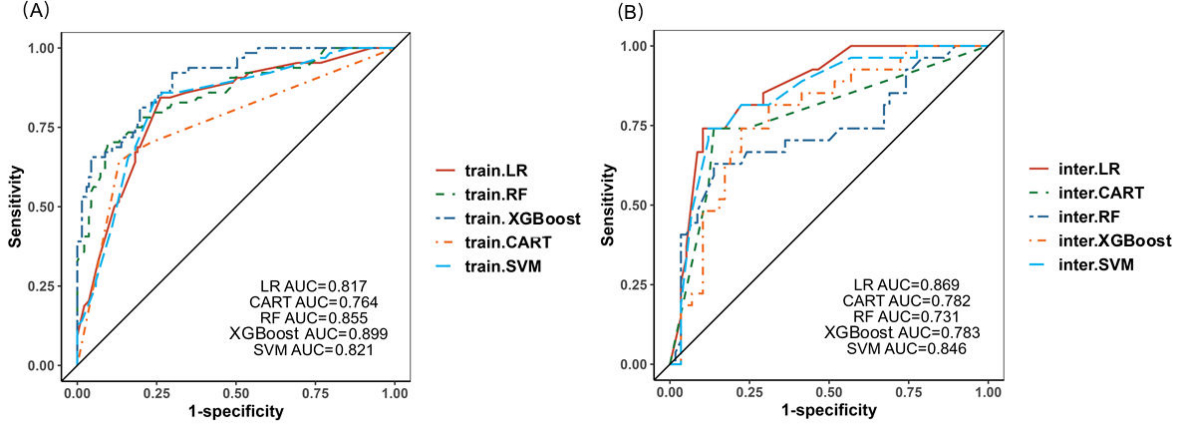
Receiver operating characteristic curves (ROCs) of each model. (A) ROCs in the training set; (B) ROCs in the validation set. AUC: area under the curve; CART: classification and regression tree; LR: logistic regression; RF: random forest; SVM: support vector machine; XGBoost: extreme gradient boosting.

The performance metrics of all 5 models are summarized as follows: Figure 7A displays the combined AUC values for the training set, while Figure 7B presents those for the validation set. Except for the CART model, the AUC values of all other models were above 0.8, demonstrating excellent discriminative ability, while the AUC values in the validation set were above 0.7. Comprehensive evaluation metrics, including AUC, accuracy, precision, recall, specificity, and *F*_1_-score, for the training set are detailed in Table 4, with corresponding validation set metrics provided in Table 5. Across all models, we observed a consistent pattern of relatively low recall coupled with high specificity.

**Table 4.** Evaluation of the 5 models in the training set.

Model	AUC^a^	Accuracy	Precision	Sensitivity	Specificity	*F*_1_-score
LR^b^	0.81	0.74	0.70	0.33	0.90	0.45
CART^c^	0.76	0.80	0.6	0.64	0.87	0.67
RF^d^	0.85	0.84	0.77	0.69	0.91	0.73
XGBoost^e^	0.90	0.85	0.82	0.66	0.93	0.73
SVM^f^	0.82	0.78	0.66	0.66	0.84	0.66

a AUC: area under the curve.

b LR: logistic regression.

c CART: classification and regression tree.

d RF: random forest.

e XGBoost: extreme gradient boosting.

f SVM: support vector machine.

**Table 5.** Evaluation of the 5 models in the validation set.

Model	AUC^a^	Accuracy	Precision	Sensitivity	Specificity	*F*_1_-score
LR^b^	0.87	0.76	0.77	0.37	0.95	0.50
CART^c^	0.78	0.78	0.68	0.56	0.88	0.61
RF^d^	0.73	0.78	0.65	0.63	0.84	0.64
XGBoost^e^	0.78	0.76	0.63	0.63	0.83	0.63
SVM^f^	0.85	0.82	0.73	0.70	0.88	0.72

a AUC: area under the curve.

b LR: logistic regression.

c CART: classification and regression tree.

d RF: random forest.

e XGBoost: extreme gradient boosting.

f SVM: support vector machine.

**Figure 8.**
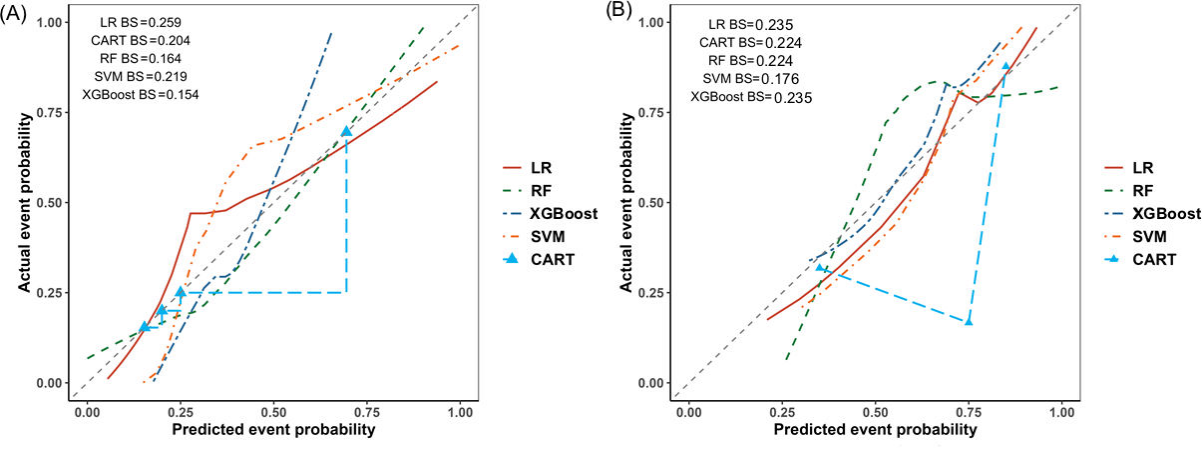
Brier index and calibration curves of the 5 models. (A) Brier index and calibration curves in the training set; (B) Brier index and calibration curves in the validation set. BS: Blair score; CART: classification and regression tree; LR: logistic regression; RF: random forest; SVM: support vector machine; XGBoost: extreme gradient boosting.

Figure 8 presents the Brier scores and calibration curves for the 5 models. Given that the CART model produces only 4 discrete predicted probabilities, its calibration curve was plotted directly using the binning method. The calibration curves for other models were generated using the Lowess smoothing method. The Brier index of the logistic regression model in the training set was relatively high (0.259). The performance of the 5 models in the validation set was worse than that in the training set. It can be seen from the calibration curve that there is a high consistency between the predicted outcomes of the models and the actual outcomes of the training set (Figure 8A). The calibration of the validation set (Figure 8B) was worse than that of the training set.

**Figure 9.**
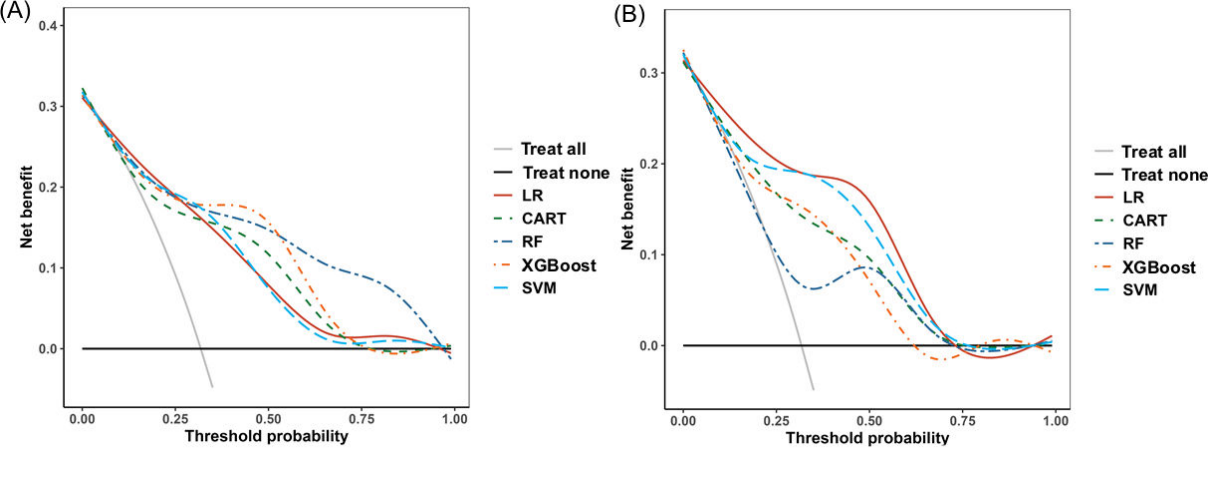
Decision curve analysis of the 5 models. (A) Decision curve analysis in the training set; (B) Decision curve analysis in the validation set. CART: classification and regression tree; LR: logistic regression; RF: random forest; SVM: support vector machine; XGBoost: extreme gradient boosting.

From the DCA (Figure 9), in the training set, the curve of the XGBoost model (at predicted probabilities of 0.8‐0.9) was slightly below the *treat none* curve, while all other models performed above both the *treat all* and *treat none* curves, demonstrating high net benefit. However, in the validation set’s DCA, the overall performance was acceptable, though slightly inferior to that of the training set. Meanwhile, the logistic regression model appeared to achieve the highest net benefit in the validation set.

Overall, the logistic regression prediction model demonstrated a high AUC; however, its overall performance in terms of recall and *F*_1_-score, as well as its calibration, was inferior to that of the other models. Both the random forest and XGBoost models exhibited an AUC difference of more than 0.1 between the training and validation sets, indicating a high degree of overfitting. The CART model offered advantages in ease of interpretation and demonstrated acceptable discriminatory power. However, due to its inherent algorithmic structure, the CART model produced a limited number of discrete risk categories (n=4), which consequently resulted in poor calibration performance.

The SVM model demonstrated good stability in AUC and DCA. However, DCA revealed its tendency to overestimate the probability of positive outcomes in negative cases and underestimate the actual risk in positive cases. It should be noted that this phenomenon in the calibration curve was not unique to the SVM model, as similar phenomena were observed in the other models. The SVM model seemed to have the best performance according to current data and analysis.

**Figure 10.**
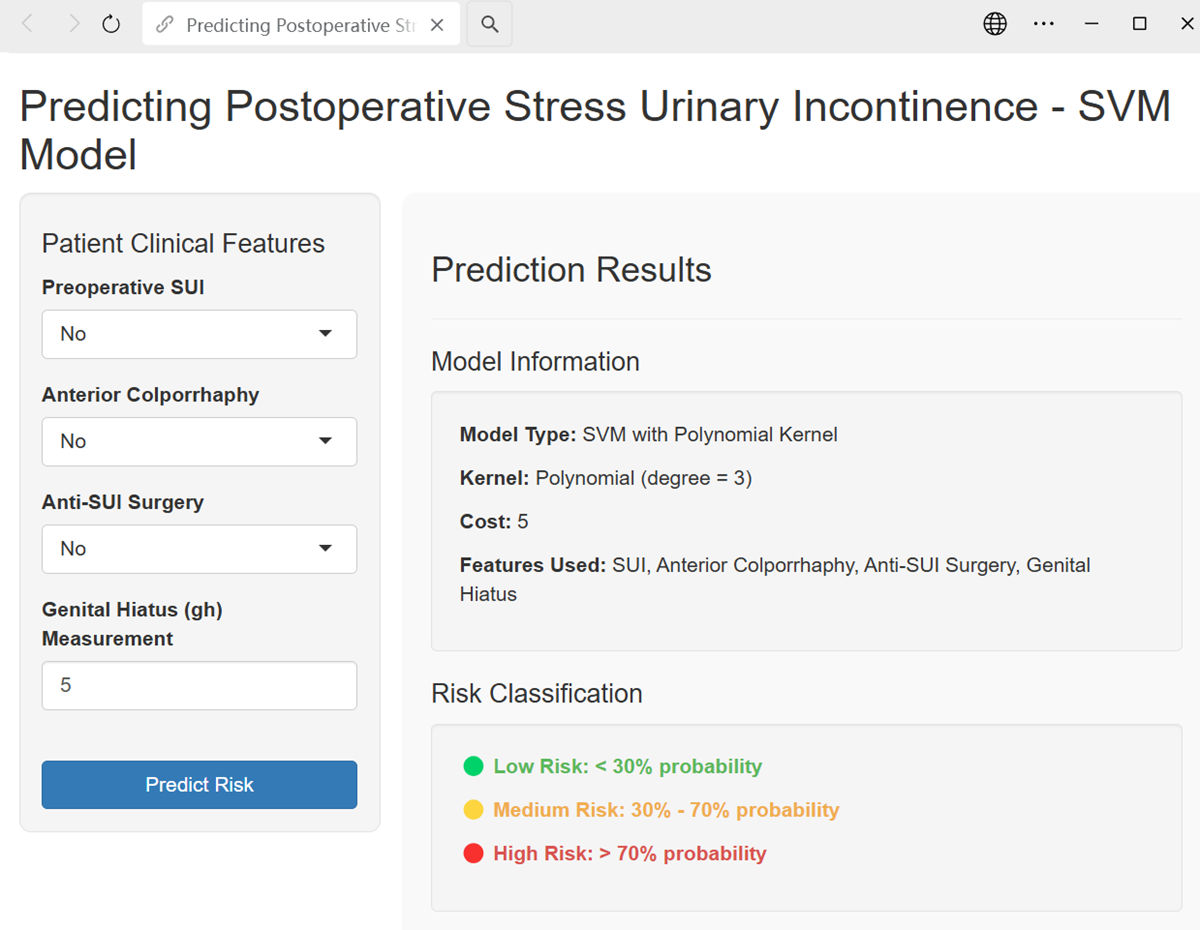
Example of support vector machine model application.

In addition to the nomogram and decision tree, we developed prediction tools for the random forest, XGBoost, and SVM models using Shiny to facilitate clinical implementation, as illustrated in Figure 10.

## Discussion

### Principal Findings

This study analyzed the data from 286 patients who underwent prolapse surgery, obtained the risk factors for postoperative SUI, and developed 5 novel prediction models, including a conventional logistic regression–based model and 4 machine learning–based models (random forest, XGBoost, CART, and SVM). Among the models, the SVM prediction model demonstrated relatively robust performance.

### Comparison With Prior Work

Jelovsek et al [9] developed the first logistic regression prediction model for SUI after prolapse surgery (termed the OPUS model) based on data from the OPUS trial, achieving an AUC of 0.72. Since the OPUS study exclusively enrolled patients without SUI, this model is only applicable to patients without SUI for predicting de novo SUI. External validation using data from the CARE (Colpopexy and Urinary Reduction Efforts) trial yielded an AUC of 0.62. When conducting external validation using available data from the CUPIDO trial, the model’s AUC was 0.63 [22]. Yasa et al [23] reported a markedly lower AUC of 0.56 during external validation with data from the Istanbul University Faculty of Medicine, concluding that the OPUS model demonstrated poor diagnostic performance.

van der Ploeg et al [10] developed the second logistic regression prediction model for SUI after prolapse surgery based on data from the CUPIDO trial (termed the CUPIDO model), achieving an AUC of 0.76. The CUPIDO-1 trial enrolled 134 patients with preoperative overt SUI, and the CUPIDO-2 trial enrolled 225 patients with occult SUI. Consequently, this model is not well-suited for patients without either overt or occult SUI preoperatively. To date, no external validation of the CUPIDO model has been conducted.

Oh et al [11] developed the third logistic regression prediction model (termed the Oh model), which is applicable to all prolapse women, regardless of preoperative SUI status or prolapse surgery type, achieving an AUC of 0.78. They partitioned data in a 4:1 ratio, allocating 20% for external validation, which yielded an AUC of 0.73.

Fu et al [14] developed the first machine learning–based predictive model for postoperative SUI following prolapse surgery by using the retrospective data of 555 patients. The study used 3 modeling approaches: random forest, XGBoost, and logistic regression. The authors determined that the XGBoost model demonstrated optimal predictive capability, with an AUC of 0.714 in the training set, 0.721 in internal validation using 5-fold cross-validation, and 0.704 in external validation with a randomly partitioned validation set. Regrettably, they only provide model parameters without the model application available.

In our study, 5 methods were used to construct the prediction model, covering tree model, linear model, and ensemble learning. It is suggested to use various methods to build a predictive model of machine learning and finally select the best model based on performance indicators [24]. Our prediction models, developed using retrospective and prospective data, are applicable to all patients with prolapse irrespective of the preoperative SUI status or prolapse surgical approach. Compared with previous random forest and XGBoost prediction models, the validation set randomly divided by our data had a higher AUC, and 2 other machine learning models were constructed: SVM and CART.

The logistic regression prediction model developed in our study demonstrated high AUC values in both the training and validation sets, though sensitivity and *F*_1_-scores were slightly lower. The low incidence of SUI after prolapse may lead to an imbalance between positive and negative samples, resulting in high specificity and low sensitivity. In contrast, both the random forest and XGBoost models exhibited notable discrepancies between the AUC of the training set and that of the validation set, with validation AUCs remaining above 0.7 yet still demonstrating signs of overfitting. As ensemble methods based on decision trees, random forest and XGBoost typically resist overfitting; however, excessive tree numbers or depth may still lead to overfitting. Despite employing various mitigation strategies, including parameter tuning, bootstrap resampling, and 5-fold cross-validation, overfitting persisted. The reproducibility of machine learning models remains a significant challenge in health care applications [25,26]. Furthermore, the observed overfitting in these models may be partially attributable to insufficient sample size. For machine learning–based predictive models, larger datasets are typically required to ensure reproducibility. Although the CART model demonstrated inferior overall performance compared to other algorithms, it showed remarkable consistency between training and validation sets. Its user-friendly nature made it more applicable in clinical work. Notably, the SVM model exhibited better performance in the validation set compared to the training set, suggesting strong potential for clinical generalization.

### Risk Factors for Postoperative SUI

Based on univariate analysis, multivariate logistic regression analysis, and Lasso regression analysis, several influencing factors of postoperative SUI were obtained: preoperative SUI, UUI, UOSUI, anti-SUI surgery, genital hiatus, and anterior colporrhaphy. SUI, UUI, and UOSUI are lower urinary tract symptoms closely related to POP. SUI is caused by the laxity of the local supporting structures of the bladder neck. In some patients undergoing POP surgery, the anatomical defects at the bladder neck remain uncorrected, leading to persistent SUI symptoms postoperatively [4]. Studies indicate that 5.2%‐31% of urinary incontinence persists after surgery [27,28]. In patients with UUI, surgery may not address the cause of UUI, and the implanted mesh and other irritative factors may even aggravate UUI. Nationwide prolapse surgery data from Finland suggested that de novo SUI occurs more frequently in patients with preoperative UUI [3].

Occult SUI refers to SUI after prolapse reduction, with an incidence of 23.5%‐42.5% [29,30]. With the progression of POP, particularly in cases of advanced anterior vaginal wall prolapse, the folding of the bladder and urethra may lead to the disappearance of SUI symptoms. After prolapse reduction, the fold of the lower urinary tract can be repaired, and the masked SUI symptoms will appear again [31]. Data from a randomized controlled trial suggested that 52% of patients with occult SUI progressed to SUI after surgery [32]. The International Continence Society, German Society of Gynecology and Obstetrics, and International Consultation on Incontinence recommend a preoperative urodynamic study to uncover occult SUI [30,33-35]. A prolapse reduction stress test for revealing occult SUI has been utilized in several predictive models [9-11].

Anterior colporrhaphy is a standard surgical procedure, just like appendectomy and tonsillectomy, and is widely performed around the world [36]. The principle of anterior colporrhaphy is based on the plication of the vesicovaginal fascia at the midline to strengthen the tissue between the vagina and bladder, previously known as the Kelly operation [37]. In the 1990s, multiple randomized controlled trials demonstrated that anterior colporrhaphy alone significantly improved SUI [38-40]. Bergman et al [38] reported an 82% cure rate for SUI following simple anterior colporrhaphy.

Genital hiatus length is defined as the distance from the external urethral meatus to the posterior edge of the hymen. An increase in genital hiatus may lead to a weakening of support for the urethra and bladder, contributing to the development of incontinence. A study about pelvic floor ultrasound and pelvic floor dysfunction found that women with urinary incontinence exhibited a longer genital hiatus [41,42]. A study by Huang et al [43] found that cystocele was associated with longer genital hiatus.

### Advantages, Limitations, and Future Perspectives

This study developed CART and SVM models for predicting SUI after prolapse for the first time, and our random forest and XGBoost models also demonstrated superior discriminative ability compared to previous models. In addition, this is the first study to develop models incorporating prospective data for SUI after prolapse surgery, thereby reducing recall bias. Our 7:3 random split of the dataset into training and validation sets for internal validation provides greater representativeness than bootstrap resampling for internal validation [22].

This study has several limitations. First, the sample size was insufficient for robust machine learning prediction, and some models still exhibited overfitting. Second, external validation using independent datasets (eg, from other hospitals or regions) was lacking. Third, the inclusion of retrospective data introduced recall bias, and the absence of objective postoperative SUI assessments, such as standardized symptom quantification and pad tests, may have led to information bias. Additionally, variability in POP-Q measurements across different clinicians over this long-term study might have contributed to measurement errors [44].

The 5 prediction models developed in this study demonstrated overall strong performance, with acceptable performance in randomly split internal validation. When considering discrimination, calibration, and clinical utility collectively, the SVM model appeared to exhibit robust performance. Based on both the current status of prediction models for SUI after prolapse surgery and this study’s limitations, we recommend: (1) the publication of experimental data, (2) the establishment of large databases from prospective studies, and (3) the standardization of the diagnosis and treatment of POP. These steps are beneficial for reducing information bias, so as to establish a generalizable prediction model with high accuracy on a national and even global scale.

### Conclusions

This study developed and internally validated 5 machine learning–based prediction models for SUI after prolapse surgery, which are applicable to all patients, regardless of preoperative SUI status and surgical approach. The model approaches included logistic regression, random forest, XGBoost, SVM, and CART. The SVM model demonstrated the most robust performance among all algorithms evaluated. We identified the following 6 predictive factors for postoperative SUI: preoperative SUI, UUI, UOSUI, concomitant anti-SUI surgery, genital hiatus, and anterior colporrhaphy. External validation is required to evaluate generalizability and determine the optimal model for clinical decision-making in postoperative SUI management.

## Abbreviations

AUC: area under the curve
CART: classification and regression tree
CUPIDO: Concomitant Surgery and Urodynamic Investigation in Genital Prolapse and Stress Incontinence: A Diagnostic Study Including Outcome Evaluation
DCA: decision curve analysis
OPUS: Outcomes Following Vaginal Prolapse Repair and Midurethral Sling
POP: pelvic organ prolapse
POP-Q: Pelvic Organ Prolapse Quantification
SUI: stress urinary incontinence
SVM: support vector machine
UOSUI: urodynamic occult stress urinary incontinence
UUI: urge urinary incontinence
XGBoost: extreme gradient boosting
